# Extracellular Matrix (ECM) Multilayer Membrane as a Sustained Releasing Growth Factor Delivery System for rhTGF-β_3_ in Articular Cartilage Repair

**DOI:** 10.1371/journal.pone.0156292

**Published:** 2016-06-03

**Authors:** Soon Sim Yang, Long Hao Jin, Sang-Hyug Park, Moon Suk Kim, Young Jick Kim, Byung Hyune Choi, Chun Tek Lee, So Ra Park, Byoung-Hyun Min

**Affiliations:** 1 Department of Molecular Science & Technology, Ajou University, Suwon, Republic of Korea; 2 Department of Orthopedic Surgery, School of Medicine, Ajou University, Suwon, Republic of Korea; 3 Department of Biomedical Engineering, Pukyong National University, Busan, Republic of Korea; 4 Cell Therapy Center, Ajou University Medical Center, Suwon, Republic of Korea; 5 Division of Biomedical and Bioengineering Sciences, Inha University College of Medicine, Incheon, Republic of Korea; 6 Lee Chun Tek Orthopedic Specialty Hospital, Suwon, Republic of Korea; 7 Department of Physiology, College of Medicine, Inha University, Incheon, Republic of Korea; University Hospital of Modena and Reggio Emilia, ITALY

## Abstract

Recombinant human transforming growth factor beta-3 (rhTGF-β_3_) is a key regulator of chondrogenesis in stem cells and cartilage formation. We have developed a novel drug delivery system that continuously releases rhTGF-β_3_ using a multilayered extracellular matrix (ECM) membrane. We hypothesize that the sustained release of rhTGF-β_3_ could activate stem cells and result in enhanced repair of cartilage defects. The properties and efficacy of the ECM multilayer-based delivery system (EMLDS) are investigated using rhTGF-β_3_ as a candidate drug. The bioactivity of the released rhTGF-ß3 was evaluated through chondrogenic differentiation of mesenchymal stem cells (MSCs) using western blot and circular dichroism (CD) analyses *in vitro*. The cartilage reparability was evaluated through implanting EMLDS with endogenous and exogenous MSC in both *in vivo* and *ex vivo* models, respectively. In the results, the sustained release of rhTGF-ß3 was clearly observed over a prolonged period of time *in vitro* and the released rhTGF-β_3_ maintained its structural stability and biological activity. Successful cartilage repair was also demonstrated when rabbit MSCs were treated with rhTGF-β3-loaded EMLDS ((+) rhTGF-β3 EMLDS) in an *in vivo* model and when rabbit chondrocytes and MSCs were treated in *ex vivo* models. Therefore, the multilayer ECM membrane could be a useful drug delivery system for cartilage repair.

## Introduction

Cartilage tissue lacks vessels through which to recruit regenerating cells after damage to its parenchyma, and thus it requires special measures for regeneration. Using the available clinical methods, bone marrow stimulation procedures and autologous chondrocyte implantations have exhibited good results after follow-up periods that are sufficient to demonstrate their efficacy [1,2]. Furthermore, the implantation of stem cells from various origins have been conducted in order to overcome the limitations of conventional cell transplantation, and many attempts have been undertaken to simplify the surgical procedures and ensure efficacy through using various biomaterials [3]. Regardless of the cell types and procedures, all cells invariably undergo steps of adhesion, proliferation, differentiation, and secretion of extracellular matrix substances at the implantation site. However, this study focuses on the harsh *in vivo* environment where the cells are implanted and on the need to modify this region in order to establish a more conducive environment for cell differentiation into chondrocytes. In particular, there is a need to support chondral regeneration in laboratory-like environments because maintaining and vitalizing differentiation is the key to successful regeneration of cartilage.

In order to establish this environment, various growth factors and pertinent cytokines have been investigated. Cartilage development is regulated by an array of growth factors including insulin-like growth factor-1 (IGF-1), transforming growth factor-ß_1_ (TGF-ß_1_), bone morphogenic protein (BMP), and basic fibroblast growth factor (bFGF) [4]. Some reports have presented the application of TGF-β_1_ for cartilage tissue engineering [5] and for the combined treatment of micro-fractures, e.g. BMP2 or BMP7 for enhancing cartilage repair [6,7].

The TGF-β family is key regulators of mesenchymal stem cell (MSC) differentiation for chondrogenesis [8]. The TGF-β cytokines control the production of extracellular matrix (ECM) through stimulating the synthesis of collagens, fibronectin, and proteoglycans [9,10]. TGF-β also has positive effects on cartilage differentiation and repair [11–13]. In contrast, TGF-β may have detrimental effects on cartilage depending on the dose and length of exposure. For example, a long exposure to high doses of TGF-β increases osteophyte formation [12]. Thus, the ability to release TGF-β in a controlled manner is crucial to the optimal application of this growth factor in cartilage regeneration. A certain delivery system should be needed to increase the delivery efficacy and bio-stability of growth factor.

The delivery system needs to provide a sustained local release of the growth factor in order to ensure its therapeutic efficacy. The advantages of a controlled release system include active ingredients being released at controlled rates over prolonged periods of time, loss of ingredients during processing could be avoided or reduced, and reactive or incompatible components could be separated. The sustained release of therapeutic drugs without degradation of the compounds and the biocompatibility of the delivery vehicle are key factors in developing an efficient and applicable drug delivery system (DDS) with limited adverse side effects. Therefore, there have been numerous reported studies that focus on controlling the release of conjugated drugs using various shapes (i.e. 3D porous scaffolds)[5], hydrogels[8], and multilayer films (e.g. chitosan, PLGA, PLLA, alginate, fibrin, etc.) [14]. Unfortunately, most current technologies encounter the possible loss of biological activity of drugs loaded during the growth factor-polymer formulation processes, which often include the use of heat, sonication, and organic solutions [15].

The multi-layering technique enables the deposition of functional polymer coatings on surfaces with variable chemistries and shapes [16]. There have been significant efforts to design multi-layer structures as coatings in order to deliver small molecules, drugs, and biomolecules from the surfaces of biomedical devices [17]. The potential of drug-loaded polyelectrolyte multilayers as anti-microbial, anti-inflammatory, and anti-cancer coatings has already been successfully demonstrated [18–20]. However, special consideration should be given to the physical dimensions and properties of the product when it is applied to locomotive organs such as joints. The thickness of the articular cartilage is several μm in animal knee joints and 3–6 mm in human knee joints. Therefore, in order to implant a delivery vehicle for the growth factor into a damaged cartilage region, the delivery vehicle’s thickness and minimal friction should be considered carefully. For narrow spaces such as joints, using technologies such as the thin membrane-based technique can be critical. In this sense, we have reported using the ECM membrane secreted by porcine chondrocytes in order to enhance the hyaline cartilage tissue formation in beagle models with cartilage defects [21]. Even though the ECM membrane is extremely thin (10–50 μm), it exhibits sufficient mechanical strength to withstand the surgical process and joint flection force after implantation. In particular, due to its lack of immune response, we successfully applied this ECM membrane in clinical use for articular cartilage repair of a micro-fracture.

In this study, we developed an ECM multilayer-based delivery system (EMLDS) that can contain the growth factor ECM membrane layer in order to deliver rhTGF-β_3_. In this work, we address the following hypothesis: using a multilayered drug delivery system, rhTGF-β_3_ is released in a sustained fashion from the EMLDS while preserving its biologic activity. Furthermore, sustained application of rhTGF-β_3_ could facilitate the differentiation of MSC and, as a result, improve the cartilage repair in the defect area. In this study, we examine the release profile of rhTGF-β_3_ followed by its biologic activity and differential potential in an *in vitro* study. Using MSCs, we confirmed that treatment with rhTGF-β_3_ embedded EMLDS ((+) rhTGF-β_3_ EMLDS) enhanced the cartilage reparability in both *ex vivo* and *in vivo* animal studies.

## Materials and Methods

### Preparation of rhTGF-β_3_-loaded EMLDS

**Fig 1:**
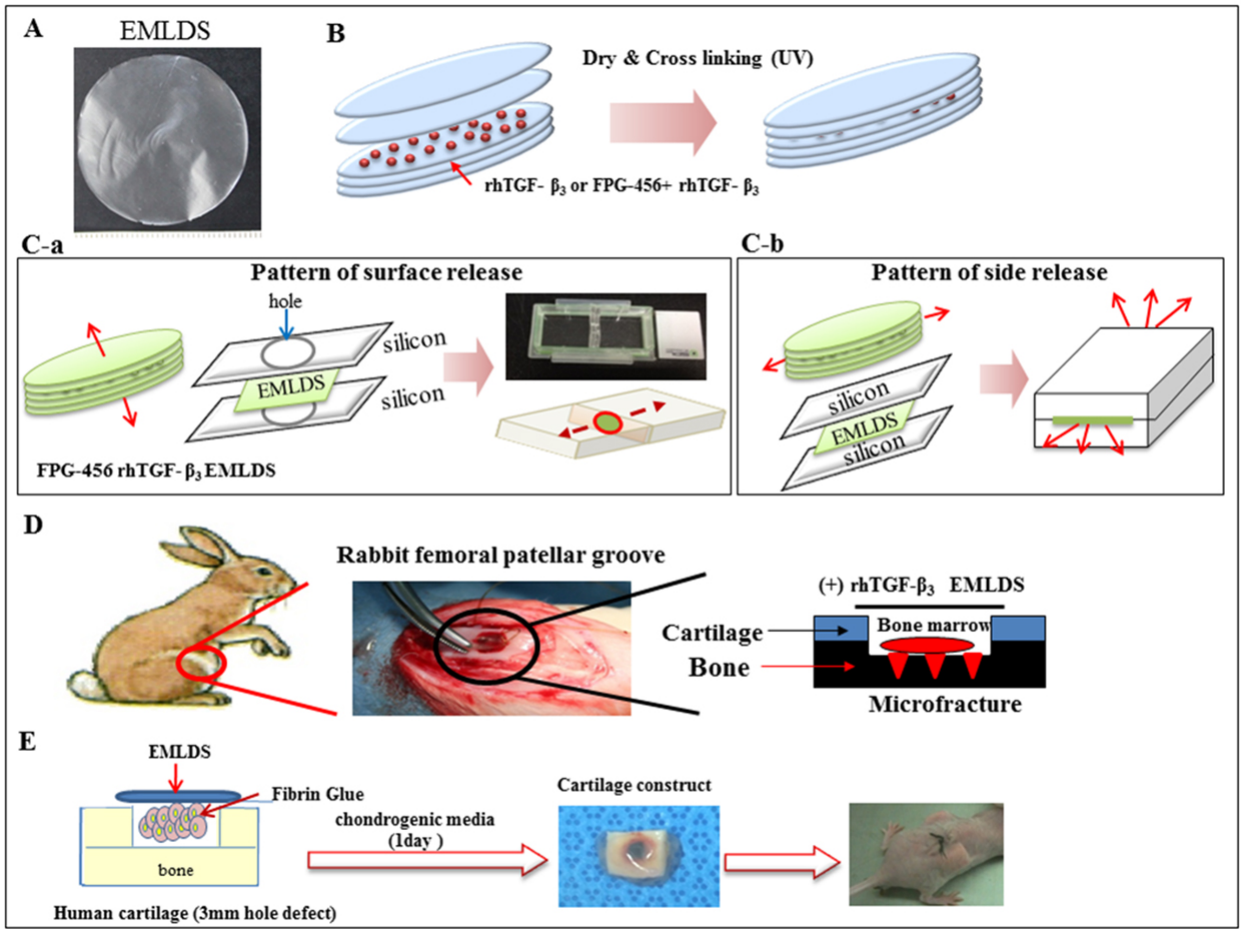
Schematic of the experiment designs and loading of rhTGF-β_3_ in the EMLDS. (A) The EMLDS was circular with a 28 mm diameter and 20 μm thicknesses. (B) Loading of rhTGF-β_3_ between the ECM membrane layers: rhTGF-β_3_ (100 ng) was loaded between the second and third layers. The rhTGF-β_3_-loaded EMLDS was dried and cross-linked using UV for 1 h in a clean bench at room temperature. (C) *In vitro* release pattern analysis using FPG-456 rhTGF-β_3_ loaded onto EMLDS. (C-a) The designed chamber that was used for pattern analysis of surface releases through wrapping the side of the EMLDS. (C-b) The EMLDS surface was covered with silicon molds in order to create an open side structure for the side release pattern analysis. (D–E) Experiment schemes of the *in vivo* surgery and *ex vivo* studies, respectively.

The ECM membrane was constructed using porcine chondrocytes as follows the our previous study [21]. In brief, chondrocytes were isolated from the articular cartilage of 2–3 week old porcine knees using 0.2% (wet/vol) collagenase (Worthington Biochemical Corp., Lakewood, NJ) in Dulbecco’s modified Eagle’s medium (DMEM) (Gibco, Invitrogen Corporation, Grand Island, NY). The primary chondrocytes were cultured at a density of 1.9×10^5^ cells/cm^2^ in 6-well plates for three weeks in order to form a cell-ECM complex membrane. To remove cell components, they underwent decellularization using sequential incubation in a 0.1% SDS solution for 2 h and then in 200 U/ml DNase solution (Sigma, USA) for 24 h at 37°C. The decelluarized ECM membrane was washed with distilled water for 1 h and pressed into a thin membrane, five of which were stacked layer-by-layer. Then, 50, 100 and 200 ng of rhTGF-β_3_ was loaded into the middle layers. Next, the rhTGF-β_3_-loaded ECM multilayer was dried and cross-linked via UV for 1 h at room temperature (Fig 1(A) and 1(B)). Only 100 ng of rhTGF-ß3 was loaded on the EMLDS because this resulted in a release profile of 10 ng/day consistent with TGF-ß3 concentrations normally used for in vitro chondrogenesis studies.

### Pattern and profile of release of rhTGF-β_3_ through the ECM multilayer

**Fig 2:**
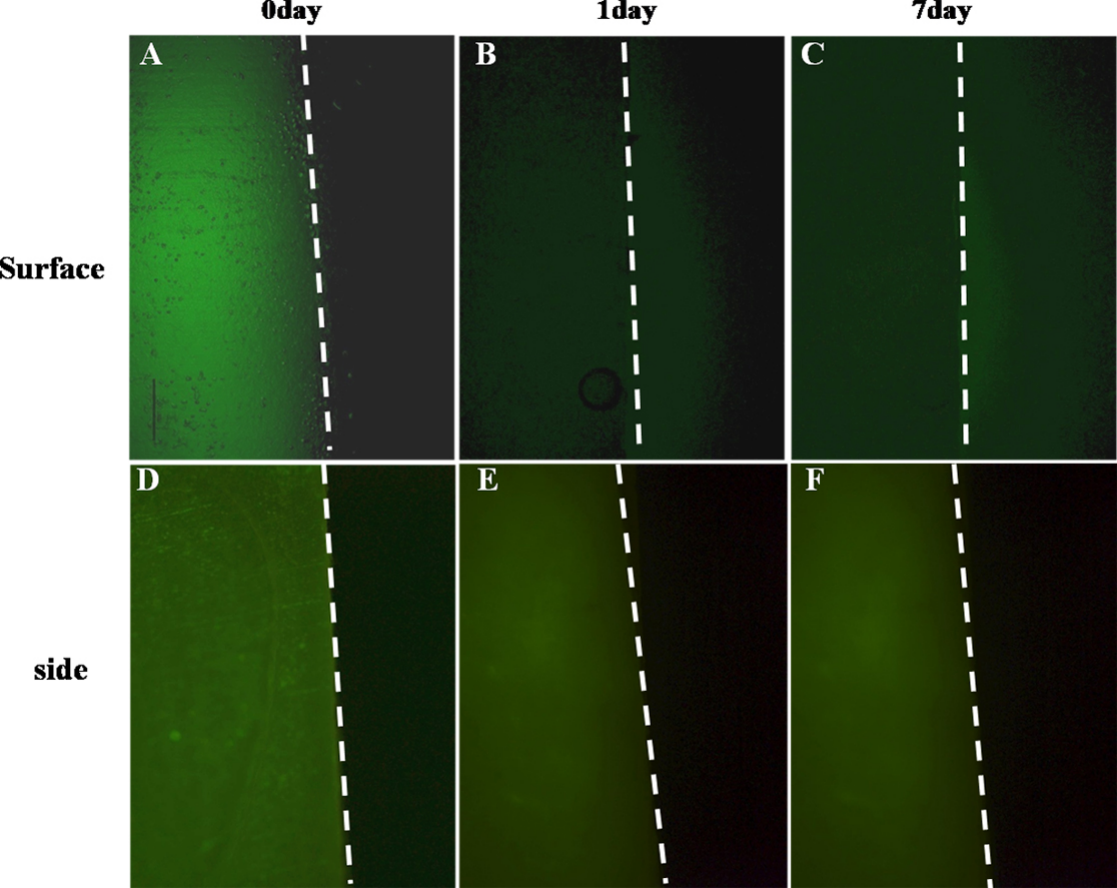
Release pattern analysis of rhTGF-β_3_ through the ECM multilayer. (A~C) The rhTGF-β_3_ release through the ECM multilayer for Day 0, Day 1, and Day 7. (D~F) The side did not exhibit rhTGF-β_3_ release. The dotted line indicates the boundary between the ECM multilayer and PBS. The loaded FPG-456 fluorescent rhTGF-β_3_ in the silicone mold is indicated in green.

In order to analyze the *in vitro* release patterns of the confined rhTGF-β_3_ from the ECM multilayer, we confirmed the release patterns through the surface and sides using green fluorescent-labeled rhTGF-β_3_ (FPG-456, Bio Acts, Korea). To begin with, specifically designed silicone molds and slide chambers were used to analyze the patterns of the surface release. Silicon molds with 1 mm thickness were punctured in order to create a hole using a 6 mm biopsy punch. The green fluorescent rhTGF-β_3_ confined in the ECM multilayer was placed between two punctured silicone molds. All edges were sealed in order to prevent the solution leaking and to prevent mold separation. It was confirmed that there was no edge leakage from the structure during incubation. Subsequently, the ECM multilayer was transferred and fixed in the slide chamber in order to observe the surface release pattern. Then, phosphate-buffered saline (PBS) was placed into the left and right chambers (Fig 1(C-a)). The release pattern was observed for seven days using a fluorescent microscope. In another experiment, the side release was tested using EMLDS-covered silicone molds with side openings, as depicted in Fig 1(C-b). All release patterns are presented in Fig 2.

In order to address the rhTGF-β_3_ release profile, the 50, 100 and 200 ng of rhTGF-β_3_-loaded ECM multilayers were incubated with 1 mL phosphate buffered saline (PBS, pH 7.2) in capped tubes and placed on a rotator at 37°C. Samples of the ECM multilayer-incubated medium were obtained at various times (1, 3, 5, 7, and 12 h; 1, 2, 3, 7, 14, 21, and 28 days). The ECM multilayers were re-incubated in 1 mL of fresh PBS after sampling. The collected samples were immediately frozen in liquid nitrogen and preserved at –80°C until quantification. The amounts of rhTGF-β_3_ released from the ECM multilayer were quantified using the corresponding ELISAs (R&D Systems, Minneapolis, USA). All samples were examined in triplicate and the data points are presented as the mean and standard deviation of these experiments. The release amount (ng) was cumulated at each time point. [22].

### Cell isolation and culture

The bone marrow MSCs and chondrocytes were isolated from New Zealand white rabbits (2 weeks old). Bone marrow mononuclear cells (BM-MNCs) were collected from aspirates of the femur by Ficoll gradient centrifugation. After washing twice with phosphate-buffered saline (PBS), cells were re-suspended in α-MEM supplemented with 10% FBS and 1% antibiotics. The MNCs were then plated 1x10^7^cells in a 150 mm dish and incubated at 37°C in humidified 5% CO_2_ air. After 6 days, the culture replenished with fresh medium to remove nonadherent cells. Chondrocytes were also isolated from same rabbit femurs cartilage. Briefly, cartilage pieces were chopped and incubated 0.1% collagenase for 12 hours. The isolated chondrocyte were plated 1x10^6^ cells in a 150 mm dish and cultured in DMEM supplemented with 10% FBS and 1% antibiotics. The second passaged MSCs and chondrocytes were used for the *in vitro* chondrogenesis, *in vivo* and *ex vivo* implantation. For the western blot analyses, the rat MSCs were isolated and cultured under conditions similar to those for the rabbit MSCs. BM-MNCs were collected from aspirates of the femurs of 7-week-old Sprague-Dawley rat by Ficoll gradient centrifugation.

### Circular dichroism (CD) analysis

A CD analysis was conducted in order to determine the deformation of the protein secondary structure during the fabrication steps of the UV cross-linking and drying for the EMLDS. A PBS solution (pH 7.4, PBS) and fresh rhTGF-β_3_ solution (1 μg/mL) were used as negative and positive controls, respectively. The EMLDS-released rhTGF-β_3_ (EMLDS-T) was collected at Day 3, Week 1, and Week 2. The rhTGF-β_3_ solution left in the PBS (PBS-T) for two weeks was also compared as a control. The conformational characteristics of rhTGF-β_3_ were measured using a Jasco-720 CD spectrophotometer (Jasco, Japan).

### Western blot analyses

In order to confirm the bioactivity of the rhTGF-β_3_ released from the EMLDS fresh solution (10 ng/mL), the PBS-incubated rhTGF-β_3_ solution (PBS-T) and EMLDS-released rhTGF-β_3_ (EMLDS-T) solution were collected. The western blot analyses were used for the cytokine activity test. The PBS-T and EMLDS-T solutions were used to treat the rat MSCs for 1 hour. The groups that were untreated and treated with fresh rhTGF-β_3_ were compared as negative and positive controls, respectively. After 1 hour of treatment with each solution, the protein was extracted using PRO-PREP (Intron, Korea). The protein (30 μg) was then separated on 8% sodium dodecyl sulfate (SDS)-polyacrylamide gel and transferred onto a nitrocellulose membrane. The primary antibodies of the rabbit anti-phospho-TGFβ-Receptor II (1:10000, Santa Cruz Biotechnology, USA), rabbit anti-phospho-Smad2 (1:1000, Cell Signaling Technology, USA), rabbit anti-TGFβ-Receptor II (1:1000, Cell Signaling Technology, USA), mouse anti-Smad2 (1:1000, Cell Signaling Technology, USA), and mouse anti-β-actin (1:4000, Calbiochem, USA) were used for the western blots. Goat anti-mouse IgM (1:2000, Calbiochem, USA) and goat anti-rabbit IgG (1:2000, Cell Signaling Technology, USA) were used for the secondary antibodies.

### In vitro chondrogenesis

The chondrogenic differentiation of the MSCs was demonstrated using a pellet culture system. The primary MSCs were passaged twice before the pellet culture experiments were performed. After washing twice with PBS, the second passaged MSCs were treated by 1x trypsin (Gibco, USA) for 10 min to recover the cells from the culture dish. The detached MSCs were suspended in α-MEM (Hyclone, USA) supplemented with 10% FBS and 1% antibiotics. Cell enumeration was determined using a hemocytometer and trypan blue dye. The aliquoted 5×10^5^ MSCs were centrifuged at 500 Xg for 5 min and the pellet was cultured in 1 mL of DMEM containing 10 ng/mL of rhTGF-β_3_ in 15 mL polystyrene conical tubes. After one day, when the cells had aggregated into a round form, the culture medium was changed to a chondrogenic medium consisting of DMEM with 100 U/mL penicillin, 100 μg/mL streptomycin, 100 μg/mL pyruvate, 40 μg/mL proline, 50 μg/mL l-ascorbicacid-2-phosphate, 1% ITS, and 100 nM dexamethasone. Then, the MSC pellets were cultured in the medium containing the 100 ng of rhTGF-β_3_-loaded ECM multilayer (MSCs+Tm). The (-) rhTGF-β3-treated (MSCs), single-time 10 ng of rhTGF-β3-treated (MSCs+Ti), and continuously 10 ng of rhTGF-β3-treated (MSCs+T) MSC pellets were used in the comparison of their condrogenesis. All pellets were harvested after four weeks.

### *In vivo* implantation for articular cartilage repair

#### Experimental design and surgical technique

The use of animals (Seventy-two New Zealand white rabbits, 16 weeks old; average weight 3.0–3.5 kg) in this experiment was approved by the Institutional Animal Experiment Committee of Ajou University, Korea (IACUC No. 2013–0013). This study was carried out in strict accordance with the recommendations in the Guide for the Care and Use of Laboratory Animals of the National Institutes of Health. All experimental details were copied from our previous studies [23,24]. An arthrotomy was made through the median longitudinal incision on the medial para-patellar with the patellar dislocated laterally in order to expose the patellar groove. A drill with a 5 mm diameter was used to create an osteochondral defect with a depth of 1.5 mm in the patellar groove. A 1 mm diameter microfracture awl was utilized for bone marrow stimulation (BMS) (Fig 1(D)).

After the BMS, the (+) rhTGF-β_3_ EMLDS was used to cover each cartilage defect. The prepared rabbits were then divided into four groups: (1) the defect group where the defect was left untreated as a negative control; (2) the BMS+m group where the subjects were covered with the (-) rhTGF-β_3_ EMLDS after the BMS; (3) the BMS+Ti group where the subjects were covered with (-) rhTGF-β_3_ EMLDS after the BMS and a single injection of 100 ng rhTGF-β_3_; and (4) the BMS+Tm group where the subjects were covered with (+) rhTGF-β_3_ EMLDS after the BMS. Normal cartilage was used as a positive control for comparison.

The membrane covering the osteochondral defects was subsequently fixed using the cross-suture method. Each knee joint was immobilized for one week after the procedure using an assistive device [24]. The cartilage was subjected to macroscopic, histological, and biochemical evaluations of the repaired tissue at Weeks 4 and 8 post-operation. The experimental designed was based on the Animals in Research: Reporting *In vivo* Experiments (ARRIVE) guidelines [25].

#### Macroscopic and histological evaluations

The rabbits were euthanized in a CO_2_ chamber following anesthesia via an injected pentobarbital (0.22 ml/kg) at 4 and 8 weeks after operation and whole distal femurs were sampled for analysis. The cartilage lesion on each patellar groove was observed by naked eye; after conducting of Safranin-O and Sirius Red staining.

#### Histological scoring

In order to diminish the effects of subjective bias, three researchers independently evaluated the quality of the repaired articular cartilage in the defects. The histological grading scale which has seven categories and 0 to 18 scores was modified from the ICRS (International Cartilage Repair Society) score [26,27] to evaluate the rabbit cartilage repair.

#### Chemical assay of repaired tissue

The samples of the repaired tissue in defects were harvested. Then a papain solution was treated for the chemical assays. In order to measure the glycosaminoglycan (GAG) content, the supernatant was subjected to a 1, 9-Dimethylmethylene Blue (DMB, Sigma, USA) solution. Chondroitin sulfate from shark cartilage (Sigma, USA) was utilized for the GAG content as a standard. The DNA content was measured using a Quant-iT PicoGreen dsDNA Reagent and kits (Invitrogen, USA) [28].

### *Ex vivo* implantation for cartilage repair

#### Experimental design and surgical technique

Human cartilage tissue was harvested from OA patients after total knee replacement surgeries at Ajou University Hospital (Korea) and Lee Chun Tek Orthopedic Clinic (Korea). Informed consent was obtained from all patients included in this study. Five groups of cartilage defect models were created as follows: (1) the defect group where a 3 mm diameter defect was made at the center of the cartilage tissue (8×8 mm) using a biopsy punch and then left untreated as a negative control, and (2) the Chondrocyte+Tm group where the seeding of the rabbit chondrocyte was followed by covering with ECM multilayer as a positive control group. The remaining groups were seeded with rabbit MSCs: (3) the MSCs+m group with no treatment followed by covering with the (-) rhTGF-β_3_ EMLDS; (4) the MSCs+Ti group treated with a single shot of 100 ng of rhTGF-β3, followed by covering with the (-) rhTGF-β_3_ EMLDS; and (5) the MSCs+Tm group which were covered with (+) rhTGF-β_3_ EMLDS.

The *ex vivo* cartilage repair model was designed based on a previously reported *ex vivo* model by Mueller-Rath [29] for this study. A mixture of 3×10^6^ cells of chondrocytes, or MSCs and blood clots, was inserted into each defect and then covered with an ECM multilayer fixed with fibrin glue (Fig 1(E)). The cartilage defect model was incubated in a chondrogenic medium for one day. Subsequently, the constructs (*n* = 6 per group) were implanted subcutaneously into the backs of 15 nude mice (6 weeks old). The use of animals in the experiments was approved by the Institutional Animal Experiment Committee of Gyeonggi Bio-Center (IACUC No. 2011-01-18). The mice were euthanized eight weeks after implantation, and each specimen was retrieved.

#### Histologic evaluation

The pellet and cartilage tissues were fixed with 10% formalin for at least 48 h. Then, they were embedded in paraffin and sectioned serially into 4 μm-thick sections using a microtome (RM2255, LEICA, Germany). The sections were stained with hematoxylin and eosin (H&E) and Safranin-O in order to evaluate their histological properties.

### Statistical analysis

Statistical analyses of the *in vivo* and *ex vivo* experimental data were conducted using one-way analysis of variance (one-way ANOVA) for multiple comparisons, and the specific inter-data differences between the mean values were identified using the Tukey-HSD test. *P* values of less than 0.05 were regarded as statistically significant and are described in the results.

## Results

### Release profile for rhTGF-β_3_

**Fig 3:**
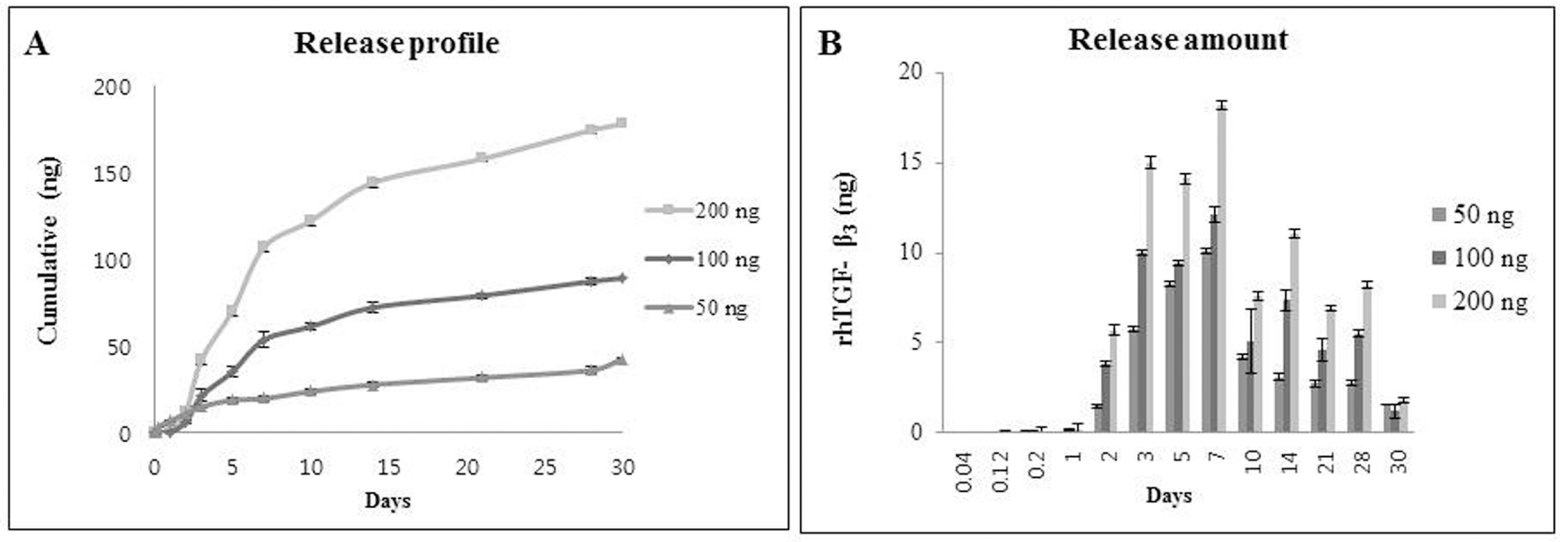
*In vitro* release profile of rhTGF-β_3_ from the EMLDS. (A) Samples incubated in PBS were collected for 30 days and the amount of released rhTGF-β_3_ was examined using ELISA. Approximately 86% of the total rhTGF-β_3_ loaded was released from the EMLDS after 28 days. (B) The release rate of rhTGF-β_3_ was approximately 8 ng/day for the first five days and approximately 4 ng/day thereafter. The release profiles did not significantly differ when the amount of rhTGF-β_3_ loaded was varied from 50 ng to 200 ng.

The final EMLDS structure was 28 mm in diameter and 20 μm in thickness (Fig 1). The release pattern was visualized using emissions of fluorescent forms of loaded rhTGF-β_3_ (Fig 2). Fig 2 presents the release pattern of rhTGF-β_3_ as it was released from the EMLDS. The left area was a green fluorescent rhTGF-β_3_-loaded EMLDS. The green fluorescent rhTGF-β_3_ passed through the surface of the ECM multilayer to the outside on Day 1 (Fig 2(B)). However, when the surface of the membrane was sealed, the green fluorescent rhTGF-β_3_ was not released through the sides, even after one week (Fig 2(D) to 2(F)). These results provide visual evidence that the rhTGF-β_3_ trapped between the membranes could only be removed through the ECM membrane. This was confirmed using the release pattern for one week because the intensity of the green fluorescence diminished over time. The rhTGF-β_3_ between the membranes was released slowly, and approximately 86% of the total rhTGF-β_3_ was released from the EMLDS over a 28-day period (Fig 3(A)). For the first 5–7 days, the rhTGF-β_3_ flowed out quickly in proportion to the time until approximately 40% of the total amount had been released. After this, the release speed slowly decreased, and only 50% of the remaining rhTGF-β_3_ was discharged. All the groups released the highest amounts of rhTGF-β3 after 1 week (~10.3, 13.2 and 18.4 ng released for groups loaded with 50, 100 and 200 ng of TGF-β, respectively). After this time the doses of TGF-β released were reduced in all the groups (Fig 3(B)).

### Circular dichroism (CD) analysis for protein structure

**Fig 4:**
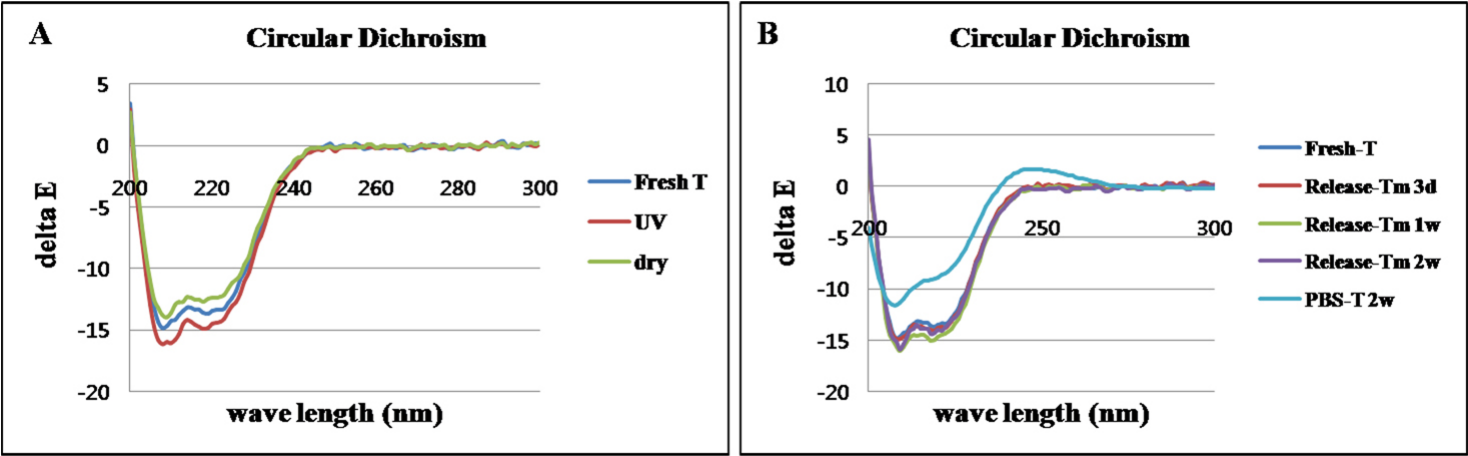
Circular dichroism (CD) analyses. (A) The EMLDS-released rhTGF-β_3_ (EMLDS-T) was not denatured after the UV cross-linking and dry process. (B) When compared with the free form of rhTGF-β_3_, the EMLDS-T did not exhibit the structural deformation. In contrast, the rhTGF-β_3_ remaining in the PBS (PBS-T) was denatured after 2 weeks.

In order to examine whether the released growth factor was denatured by the UV treatment and drying methods, the activity of the PBS-incubated rhTGF-β_3_ (PBS-T) and the EMLDS-released rhTGF-β_3_ (EMLDS-T) was assessed using CD analyses. Fresh rhTGF-β_3_ was also analyzed as a positive control. The structures of all groups had the same patterns of CD spectra, except the PBS-T. This result demonstrated that the rhTGF-β_3_ in the EMLDS was not denatured by the UV cross-linking and drying processes (Fig 4(A)). When the released rhTGF-β_3_ was compared with the fresh rhTGF-β_3_, the protein structures of the EMLDS-T retained their intact form for two weeks. However, the PBS-T exhibited significant degeneration in the protein structure at two weeks (Fig 4(B)).

### Bioactivity assay for rhTGF-β3

**Fig 5:**
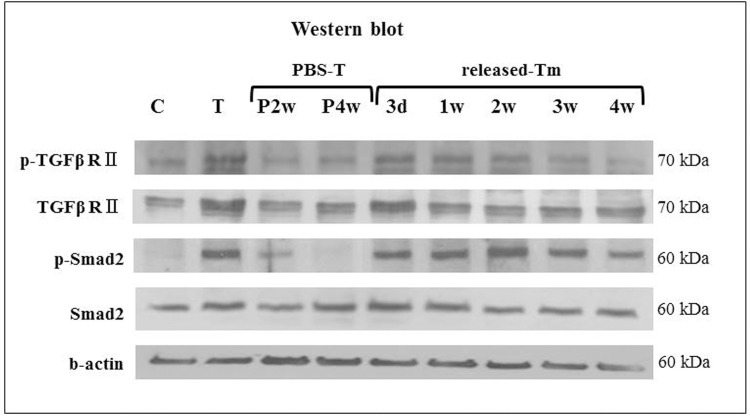
Bioactivity assays of rhTGF-β_3_ using western blots. C: untreated; T: treatment with fresh rhTGF-β_3_; PBS-T: treatment with PBS-incubated rhTGF-β_3_; and EMLDS-T: treatment with EMLDS-released rhTGF-β_3_. The assays for the Day 3 and Week 2 EMLDS-T clearly demonstrate the phosphorylation of TGF-β_3_ RII and the downstream molecule of Smad2.

In order to confirm the EMLDS-T bioactivity, the phosphorylation of two types of signaling receptors was examined: TGF receptor II and Smad2. The experimental groups were subjected to various treatments of rhTGF-β_3_ (fresh, P, and Tm). While the untreated control group did not up-regulate the phosphorylation of the signaling receptors, the fresh rhTGF-β_3_ treatment could phosphorylate the TGF receptor II and Smad2. The sustained cytokine treatment with EMLDS-T also exhibited dominant phosphorylation of TGF receptor (R) II and Smad2, compared with the untreated controls. In particular, the treatments for Day 3 and Week 2 in the EMLDS-T could clearly phosphorylate the TGF-β_3_ RII and downstream molecules of Smad2. However, the treatment at Weeks 2 and 4 PBS-T did not phosphorylate the signaling receptors (Fig 5).

### *In vitro* chondrogenesis

**Fig 6:**
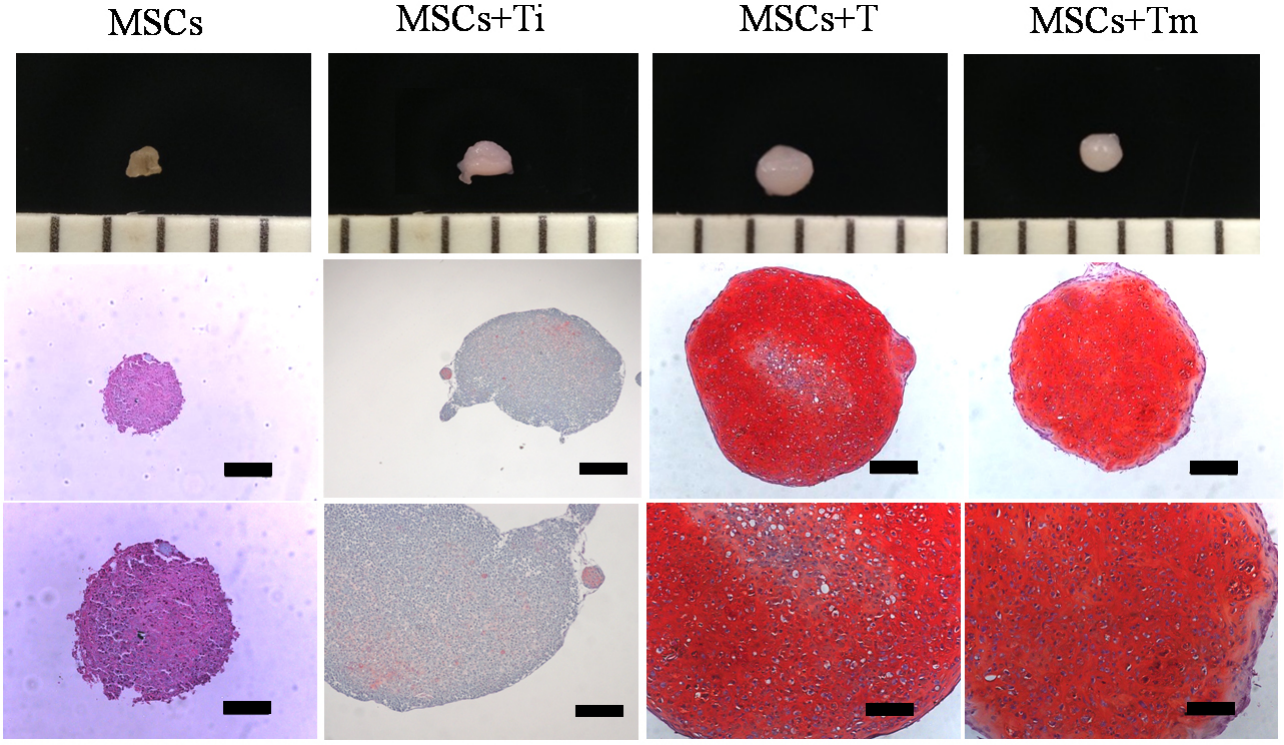
Safranin-O staining for chondrogenic differentiation of *in vitro* pellet culture at Week 4. MSC+Ti: single treatment of rhTGF-β_3_; MSC+T: continuous treatment of rhTGF-β_3_; MSC+Tm: treatment of (+) rhTGF-β_3_EMLDS. Scale bar: upper row = 200 μm, lower row = 100 μm.

The effect of the released rhTGF-β_3_ on the chondrogenic differentiation was assessed histologically after four weeks in the pellet culture. Fig 6 depicts the histological conditions of the pellets after four weeks, when the cytoplasm was stained with fast green/Safranin-O and the negatively charged GAGs were stained red. The positive effects on the cartilage formation of releasing the rhTGF-β_3_ were clearly confirmed at four weeks. While the (-) rhTGF-β_3_ (MSCs) and single-time rhTGF-β_3_-treated (MSCs+Ti) groups did not have GAG formation, the rhTGF-β_3_ released from the EMLDS-treated (MSCs+Tm) and continuously rhTGF-β_3_-treated (MSCs+T) groups exhibited well synthesized chondrogenic GAGs. This cell pellet model revealed that the rhTGF-β_3_ released from the EMLDS exerted biological effects on the chondrogenesis of MSC and could enhance cartilage formation.

### Rabbit articular cartilage repair

#### Gross findings and histological evaluation of cartilage defects

**Fig 7:**
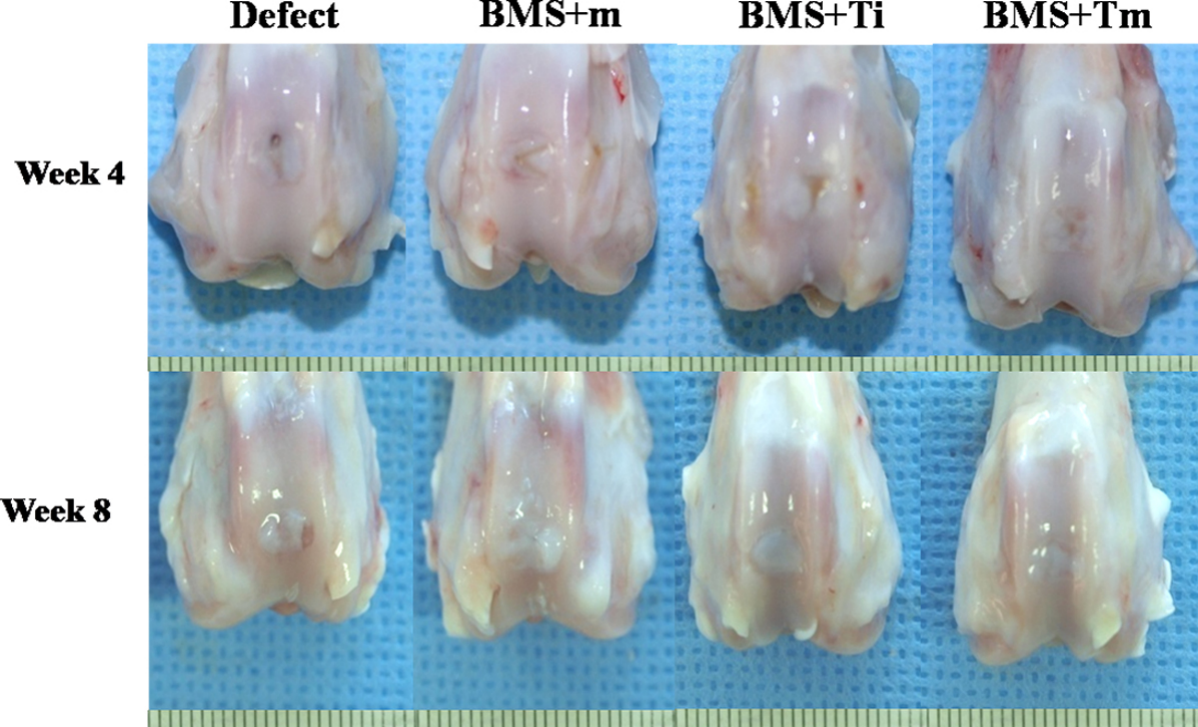
Gross observations of the repaired cartilages. In the Week 4 samples, the BMS+Tm group exhibited a smooth glistening appearance, and continuity with the surrounding host cartilage tissue was also observed. In contrast, the defect was not repaired in the defect group and was partially filled with fibrous tissues in the BMS+m and BMS+Ti groups. At Week 8, the white glistening appearance of repaired tissues was seen in all groups. BMS+m: Covered with the (-) rhTGF-β_3_ EMLDS after the bone marrow stimulation; BMS+Ti: Covered with (-) rhTGF-β_3_ EMLDS and a single injection of rhTGF-β_3_ after the bone marrow stimulation; BMS+Tm: Covered with (+) rhTGF-β_3_ EMLDS after the bone marrow stimulation.

In the four-week samples, the BMS+Tm group exhibited a smooth, glistening appearance and continuity with the surrounding host cartilage tissue was also observed. In contrast, the defect was not repaired in the defect group, and it was partially filled with fibrous tissues in the BMS+m and BMS+Ti groups. At eight weeks, the white glistening appearance of the repaired tissues was visible in all groups. Although the defects of all groups were filled with repaired tissue, only the defect area of the BMS+Tm group was filled with cartilage that appeared normal (Fig 7).

**Fig 8:**
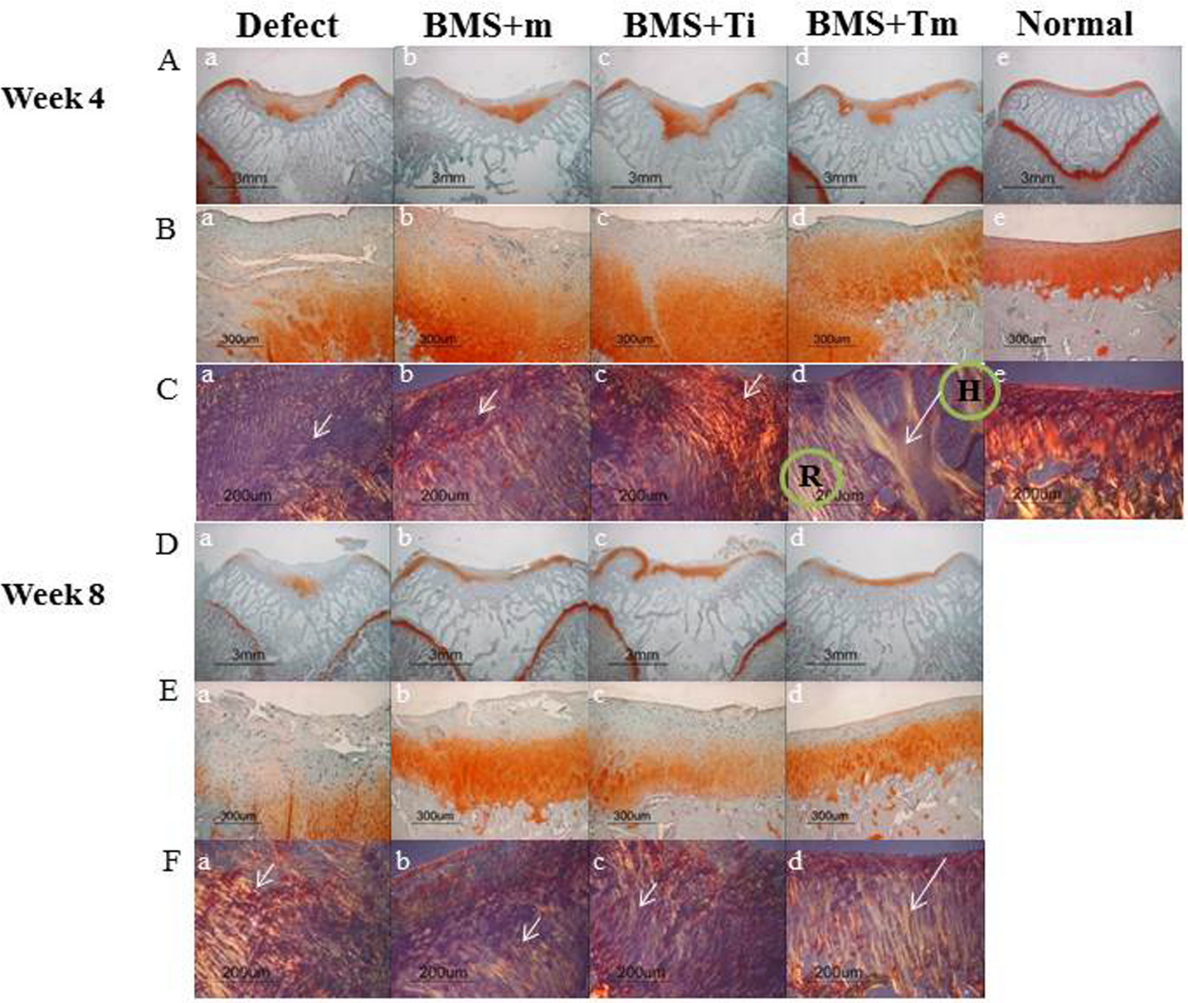
Histological analyses of the repaired cartilage at Week 4 (AC) and Week 8 (D-F). Safranin-O and Sirius red staining images of normal cartilage were provided as a control (A-e, B-e, C-e). The defect of fibrous/hyaline cartilage was regenerated in all groups at Week 4. Only BMS+Tm exhibited regeneration tissue as unnormal hyaline cartilage tissue at Week 8 (A-a to D-d). The repaired tissue in BMS+Tm was well organized with intense ECMs; furthermore, the cell distribution was composed of columnar and cluster cells with a hyaline character, even though its surface and cartilage bone were irregular (A-b to E-d). In the Sirius red staining, the collagen fibers were not oriented in the defect, BMS+m, and BMS+Ti groups (short arrows). However, the collagen fibers were more organized and integration was observed between the repaired cartilage (R) tissue and host cartilage (H) in the BMS+Tm group (long arrows; A-c to F-d).

At Week 4, fibrous hyaline cartilage occupied the defect in the Safranin-O staining of all groups. However, its surface was rough and the cells generally lacked orientation in the defect, BMS+m, and BMS+Ti groups. At Week 8, the defect was partially filled with fibrous tissues in the defect group, and it was occupied by a significant amount of fibrous tissue covering the articular surface in the BMS+m and BMS+Ti groups. However, the BMS+Tm group exhibited repaired tissue that appeared to be normal hyaline cartilage tissue. Hyaline cartilage tissues with mature matrices and columnar organizations of chondrocytes were also observed in the BMS+Tm group (Fig 8(A-a) to 8(D-e)). The repaired tissue was well organized with intense ECMs. Furthermore, the cells were columnar and clustered as seen in hyaline cartilage (Fig 8(A-b) to 8(E-d)).

When stained with Sirius red, the collagen fibers were disoriented in samples from the defect, BMS+m, and BMS+Ti groups at Week 4 (Fig 8(A-c) to 8(C-c); short arrows). In contrast, the collagen fibers were more organized and integration was observed between the repaired cartilage (R) tissue and host cartilage (H) in the BMS+Tm group (Fig 8(C-d); long arrow). At Week 8, the oriented arrangements of the collagen fibers were not observed in the defect, BMS+m, and BMS+Ti groups (Fig 8(F-a) to 8(F-c); short arrows). In the BMS+Tm group, well-oriented arrangements of collagen fibers similar to normal cartilage were found at eight weeks. Furthermore, the integration between the repaired tissue and host tissue was also observed (Fig 8(F-d); long arrow).

**Fig 9:**
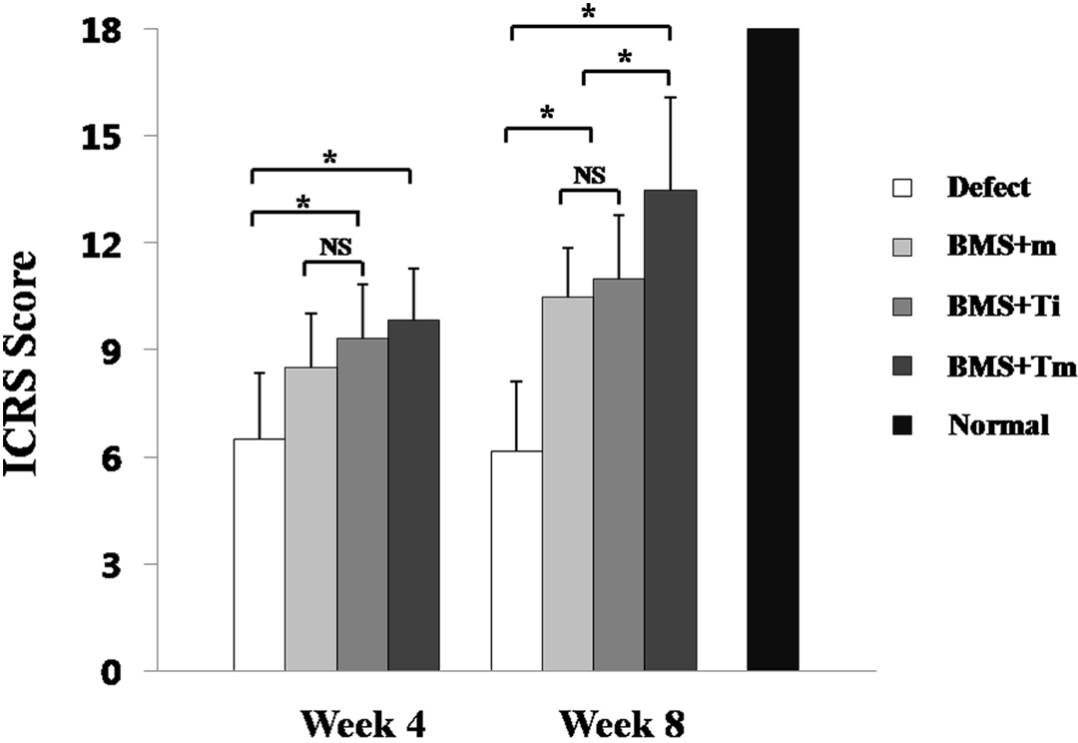
ICRS repaired cartilage scores at Week 4 and Week 8. The total ICRS histological score increased significantly with time in all groups, except the defect group. While no significant difference was observed between the BMS+m, BMS+Ti, and BMS+Tm groups at Week 4, a significant difference was exhibited in the ICRS score at Week 8. (N = 6; Error bars indicate the standard deviation; *p<0.05)

The ICRS histological score confirmed a significant improvement with time in all but the defect group. It was not until Week 8 that the BMS+Tm, BMS+Ti, and BMS+m groups showed differences in ICRS scores (femoral condyles (N) = 6; BMS+Tm and BMS+Ti were *p<0.05, while BMS+Tm and BMS+m were *p<0.05; Fig 9).

#### Chemical assay of the repaired tissue

**Fig 10:**
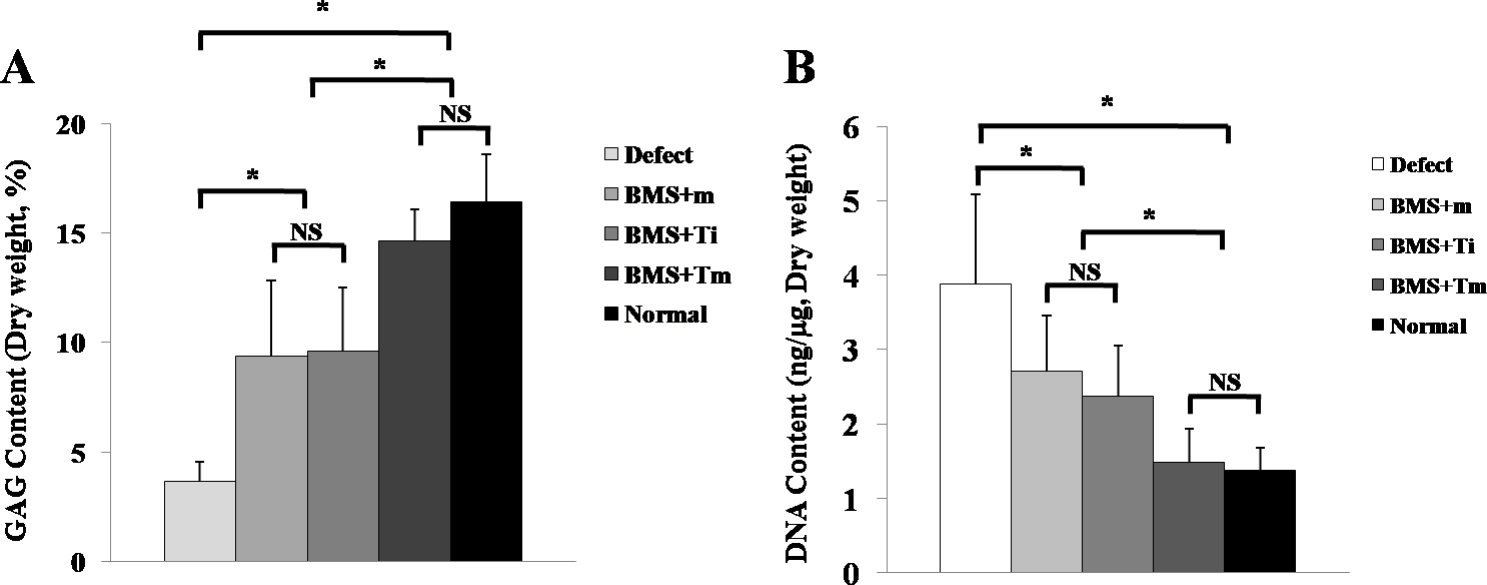
Chemical assay of repaired cartilage. (A) Changes and comparison of the GAG content among the experimental groups at Week 8. The GAG content in the BMS+Tm group was higher than that of the defect, BMS+Ti, and BMS+Tm groups. Moreover, they were significantly different in the statistical analyses (N = 6; *p*<0.05). The BMS+Tm group did not differ from that of the normal tissue in the statistical analysis (N = 6). (B) Changes and comparison of the DNA content among the experimental groups at Week 8. While the level of DNA content in the BMS+Tm group was significantly lower than that of the defect, BMS+m, and BMS+Ti groups in statistical analysis (N = 6; *p*<0.05), it did not exhibit a difference compared with the normal tissue. (N = 6; Error bars indicate the standard deviation; *p< 0.05)

The BMS+Tm group showed a significantly higher GAG amount than the defect, BMS+m, and BMS+Ti groups. However, there was no statistical difference in the GAGs amount of the BMS+Tm group and normal cartilage at Week 8 (Fig 10(A)). In the BMS+Tm group, lower levels of DNA content (similar to normal cartilage) were also detected compared with the defect, BMS+m, and BMS+Ti groups. Taken together, these results demonstrate that the repaired cartilage of the BMS+Tm group was comparable to hyaline cartilage (Fig 10(B)).

### *Ex vivo* implantation for human cartilage repair

**Fig 11:**
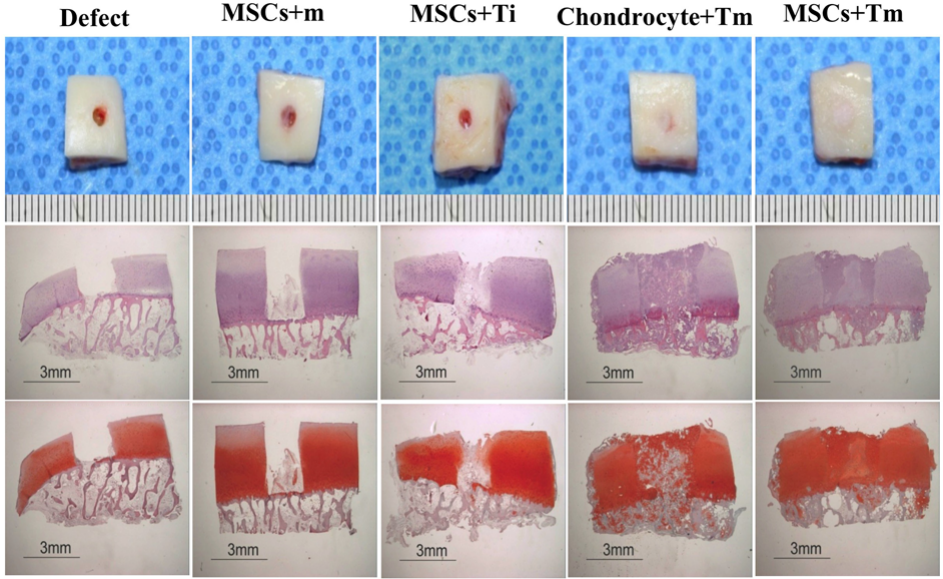
Histology of the repaired tissue in the cartilage defects. The effect of (+) rhTGF-β_3_ EMLDS was examined in the repair of the cartilage defects after eight weeks of *ex vivo* implantation. The H&E (second line) and Safranin-O (third line) stained images demonstrate that MSCs+Tm group exhibited complete healing of the defect with hyaline-like cartilage. As a reference, the MSCs+Ti group completely filled the defect but did not exhibit cartilaginous phenotype using Safranin-O strain. The defect group did not fill the defect and the MSC group also exhibited repair of fibrotic tissues. (Ruler scale = mm Scale bar = 3 mm)

**Fig 12:**
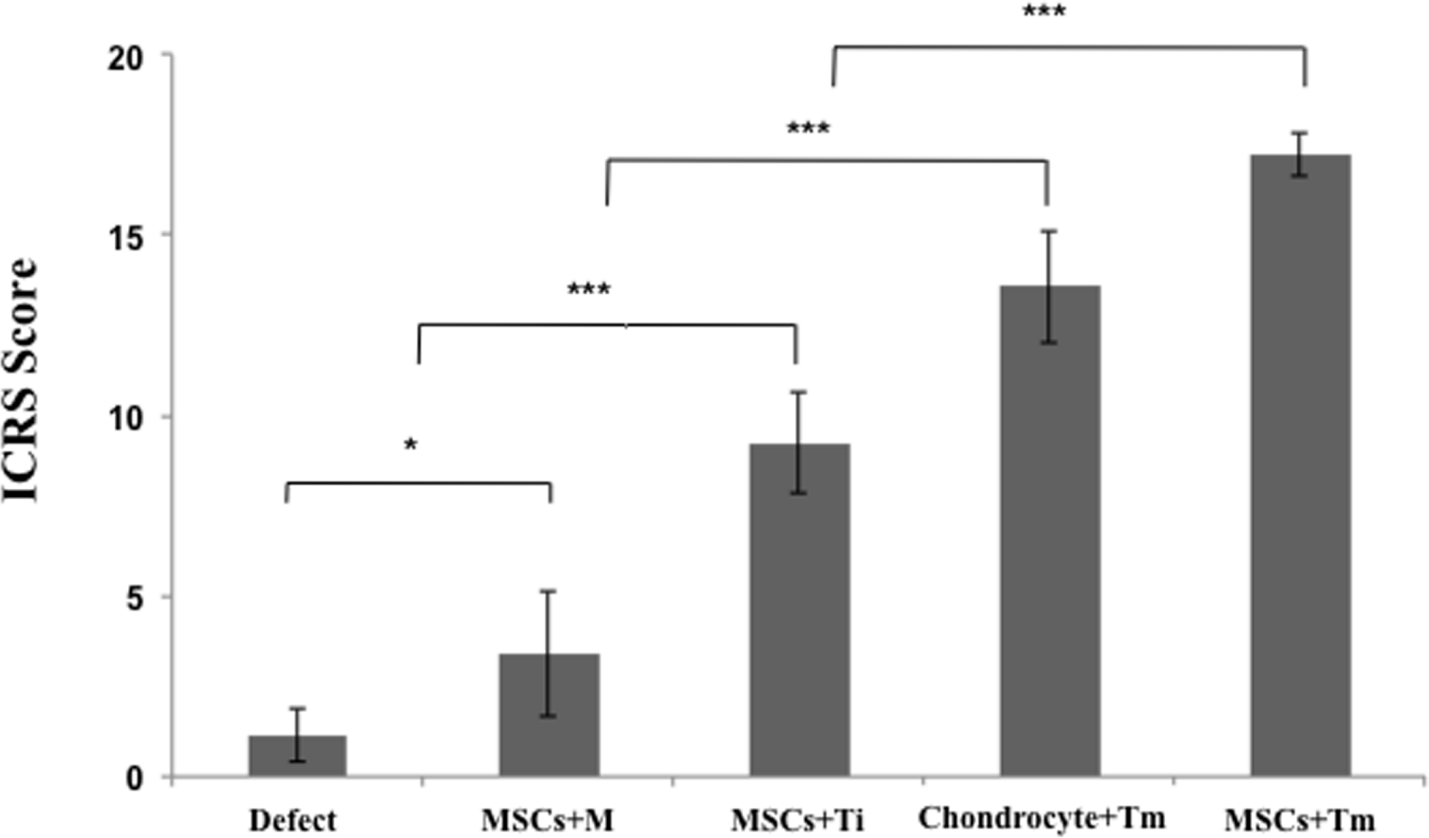
ICRS scores at eight weeks post-surgery based on the *ex vivo* histology. In all groups, there was a statistical increase in the cartilage regeneration in the defect area. In particular, the MSCs+Tm group exhibited the highest ICRS score of cartilage tissue regeneration among the groups.

All cartilage defect models, which mimicked human cartilage defects, were transplanted subcutaneously into the backs of nude mice for cartilage regeneration. The defect area of the defect group remained empty. In the positive control group implanted with rabbit chondrocyte, the defect area was filled; however, the Safranin-O staining only indicated localized staining on the upper portion of the repaired tissue. Integration with the marginal host cartilage was good. In the rabbit MSC+m group where only MSCs were injected into the defect area, the defect area was partially filled with fibrous tissue at the bottom. In the MSCs+Ti group where MSCs were injected with subsequent single shot of rhTGF-β_3_ into the defect area, unlike the MSCs+m group, the defect area was found mostly filled with fibrous tissue, not cartilage, as demonstrated using the Safranin-O staining. In the MSCs+Tm group, for which rhTGF-β_3_ was constantly released, the defect area was completely filled with cartilage tissue demonstrating perfect integration with the marginal cartilage. As stated above, the MSCs+Tm group exhibited the best cartilage repair and it even demonstrated superiority to the positive control group (Fig 11). The ICRS histological score confirmed a significant improvement in 3 groups (MSCs+Ti, Chondrocyte+TM and MSCs+TM) more than defect and MSCs+M groups (Fig 12).

## Discussion

In this study, we developed rhTGF-β_3_-loaded EMLDS and evaluated the biological effect of the rhTGF-β_3_ released from the EMLDS. The results demonstrated that the EMLDS released rhTGF-β_3_ at an appropriately sustained speed and that the released rhTGF-β_3_ maintained its biological functions under laboratory conditions causing improved chondrogenesis by MSC and an enhanced cartilage repair potential for the MSC in both *in vivo* and *ex vivo* models.

The multi-layering technique has an advantage that allows for the precise control of various parameters, such as thickness, chemistry, and mechanical properties [30]. The protein adsorbed or embedded in the multilayered films, such as FGF, has been demonstrated to retain its activity [4]. This technique was also examined as a controlled release system for the growth factor. For example, Jessel et al. demonstrated that the rhBMP-2 and TGF-β_1_ embedded in a multilayered architecture containing b-cyclodextrins could synergistically induce embryonic stem cells to differentiate in cartilage and bone [31]. Furthermore, Nisarg et al. reported the release regulation of VEGF and rhBMP-2 using different numbers of layers in the multilayer film [32].

We similarly confirmed that the growth factor release from the ECM membrane was regulated by the number of layers. The five-layer ECM membrane (approximately 100 μm thick) exhibited good handling properties for *in vivo* implantation in this pilot study. In addition, the five-layer ECM membrane did not exhibit differences in the rhTGF-β_3_ release profile compared with the ten-layer ECM membrane. Based on these results, we developed a method of producing the laminated multilayer as a delivery system of the growth factor; this multilayer combined an ECM membrane, a drug-carrying layer, and a barrier membrane; the rhTGF-β_3_ was loaded between the second and third layers.

The use of EMLDS has a number of advantages. First, many methods use organic solvents but this membrane did not require them. This is important for biomolecules such as nucleic acid and proteins, which have limited solubility in non-aqueous solutions and are susceptible to denaturation. The rhTGF-β_3_ loaded into the EMLDS was slowly released for approximately one month with approximately 90% of the amount loaded released *in vitro*. Second, the fabrication process of the EMLDS is relatively simple. Only a short time is required for drying and UV cross-linking while going through the lamination process and trapping the drug between the layers. This processing did not denature, and it exhibited slow structural degeneration of the rhTGF-β_3_ being released, as seen in the CD analyses. Furthermore, we investigated the bioactivity of the EMLDS-released rhTGF-β_3_ and demonstrated that it maintained its biologic activity based on the western blot analysis results that the TGF-β receptor II and Smad2 were phosphorylated by the released rhTGF-β_3_. Because the TGF receptor II and Smad2 are signaling receptors that form heteromeric cell surface complexes with the rhTGF-β_3_ as one of the earliest events in the cellular response to multifunctional growth factors, their phosphorylation indicates the successful beginning of the rhTGF-β_3_ stimulation on the effector cells [33–35].

From a material perspective, the EMLDS provided additional benefits for the repair of cartilage defects after the bone marrow stimulation, beyond serving simply as a physical barrier. Its advantageous properties (e.g. permeability, biodegradability, and strength) have already been demonstrated in our previous study [21]. In particular, the 30–60 μm ECM membrane exhibited a tensile strength of more than 85 N. The permeability of the ECM membrane also exhibited a diffusion coefficient that was sufficient to deliver small molecules from the synovial fluid into the repaired tissue. By extension, the EMLDS in the current study did not exhibit rhTGF-β_3_ release through its sides but only from its surface. This type of release pattern could be explained through the solidity of the densely laminated side structure generated after the UV cross-linking. This method, based on the multi-layering confinement technique, may be superior to other methods for the delivery of growth factor. For example, the release profile from simply doused growth factor appeared faster with more than 90% being released within one week [36–38]. For comparison, five EMLDS exhibited sustained release of rhTGF-β_3_ over 30 days in this study. The targeted 10 ng release profile of rhTGF-β_3_ was achieved from a five-layered membrane loaded with 100 ng of rhTGF-β_3_. The five-layered membrane (20 μm) used in this study was chosen after consideration of its sustained release performance, good handling properties, and potential applications in the medical industry.

In order to ensure appropriate implantation of the drug delivery system used for articular cartilage, it should morphologically fit the damaged region. Cartilage from mice, which are the most commonly used experimental animals, is 100–200 μm thick, while pig cartilage is 1–2 mm thick. Human articular cartilage is 3–6 mm thick. Therefore, for the implant material to be inserted into the damaged region without affecting the joint motion, its thickness should be as close to this range as possible. Furthermore, bone marrow stimulation and cell transplantation, which are popular procedures for cartilage repair, need extra space for blood clots, including the endogenous bone marrow stem cell, or for cultured cells to be injected. Therefore, a multilayer membrane system is appropriate for application in such thin cartilage defects. Other methods that might be applied in shallow cavities could include soluble, adherent, and self-gelling carriers [39]. Kuo used collagen sponges and, even though it was successful when press-fitted into the hole via microfracture, the sponge covering the cartilage defect failed to attach [6].

The *in vitro* pellet culture study is a direct example demonstrating that the continued supply of rhTGF-β_3_ is an essential condition for the MSC pellet formation and chondrogenesis. In the untreated and single treatment rhTGF-β_3_-treated MSC+Ti groups, the pellets were not differentiated into cartilage with their size being very small and the tissue being fibrous. In contrast, for the continuously treated MSC+T and the EMLDS rhTGF-β_3_-treated groups there was good chondrogenesis. As mentioned above, the rhTGF-β_3_ released from the EMLDS was determined to have maintained its activity and supported the chondrogenesis of the MSCs. In the same manner, without losing its activity within the membrane, the rhTGF-β_3_ sustained release system differentiated the MSCs as effectively as the *ex vivo* study.

The data from the CD analyses, western blots, and chondrogenesis of MSC by pellet culture confirmed the effect of the constantly released rhTGF-β_3_ on the differentiation of MSCs into chondrocytes, which indicated the presence of biologic activity. Based on these results, stem cell implantation was designed for *in vivo* experiments using a microfracture rabbit cartilage model. Microfracture for the endogenous stem cell induction is the most highly recommended clinical technique, and stem cell implantation is a method of implanting exogenously cultured cells. These two methods are surgical operations that are being widely investigated. We attempted to prove that the EMLDS induced effects in both endogenously and exogenously produced stem cells.

The MSC induced from the subchondral bone via microfracture is known to be significant in chondral regeneration [24,40,41]. We have reported that the MSCs are drained from the bone marrow after microfracture procedures and that the results vary according to the size and number of holes in the subchondral bone [42]. As a result of previous *in vivo* experiments, simply covering the microfracture area with a membrane to preserve MSC is known to be beneficial to chondral repair [21]. The benefits of coverage with the EMLDS in the current study were consistent with our previous results. In the histological and biochemical analyses, the BMS+m group exhibited statistically higher synthesis of GAGs than the defect group, but with a poorly organized collagen matrix. This appeared to result from the ability to maintain cells at the defect site due to the membrane coverage [21]. The BMS+m and BMS+Ti groups did not exhibit statistical differences. However, it is important to note that the BMS+Tm group exhibited significant differences compared with the BMS+m and BMS+Ti groups at eight weeks, and it exhibited GAG formation similar to normal cartilage tissue in the histological and biochemical analyses.

As a complementary experiment, an *ex vivo* study was used to analyze whether (+) rhTGF-β_3_ EMLDS was effective in human cartilage regeneration compared with conventional cell implantation procedures. The multilayer was tested after forming chondral defects on the osteochondral fragments extracted from the patient and injecting MSCs into the defective region. The cartilage defect model filled with cells was implanted subcutaneously into a nude mouse. MSC groups with or without a single shot of rhTGF-β_3_ treatment failed to induce MSC differentiation into chondrocytes, which resulted in the formation of mostly fibrous tissue. As expected, the chondrocyte implantation as a model of ACI (positive control) exhibited successful cartilage repair. More interestingly, the MSC implantation based on the constant stimulation of rhTGF-β_3_ exhibited significantly better cartilage generation than that of the positive control group. The new tissue only slightly differed to the surrounding normal cartilage and the integration was quite good. Taken together, all results in this study demonstrated that the rhTGF-β_3_ delivery using the EMLDS had a positive effect on MSCs. Both the endogenous stem cell therapy (microfracture) and exogenous stem cell implantation resulted in excellent chondral regeneration.

Until now, MSC treatment has not demonstrated improved results compared with conventional autologous chondrocyte implantation techniques; however, this may be overcome by applying the technology developed in this study. One shortcoming of this study was that no long-term follow-up has been undertaken yet for cartilage repair after implantation. A relatively short follow-up of eight weeks was undertaken, because the loaded rhTGF-β_3_ in the EMLDS was mostly released by Week 4. Furthermore, the role of rhTGF-β_3_ is an induction of the MSC differentiation at a relatively early period of implantation.

The induction of MSCs to form chondrocytes in the early stages after the microfracture procedure is very important. Thus, if successful differentiation of MSCs is achieved with the secretion of the chondrocyte extracellular matrix, it is thought to be a favorable environment for the formation of mature cartilage. In accordance with this rationale, the efficacy of the rhTGF-β_3_ released from the EMLDS to induce chondrogenesis was tested using the *ex vivo* model in the current study in order to mimic human cartilage environments. In the results, good integration between the host and generated tissues was demonstrated in the (+) rhTGF-β_3_ EMLDS group (MSCs+Tm). However, the influences of rhTGF-β_3_ delivered using the EMLDS on the implanted MSC were not verified in real joints in this study. The current modified *ex vivo* experiment, i.e. subcutaneous implantation of human osteochondral fragments into nude mice, was favorably selected before entering large animal or human experiments in order to verify the efficacy of the implanted biomaterials and cells. This study utilized an *ex vivo* model that was developed to closely reflect the physiological cartilage environment with contact to all sides of the intra-articular regions, even though it was not a real cartilage defect. Previous reports have demonstrated the potential of the nude mouse osteochondral *ex vivo* model for demonstrating integration of the implanted tissue and regeneration of the cartilage [29,43]. Another limitation of the current *ex vivo* study is that the cultured chondrocytes and MSCs could not be prepared in autologous cells because this experiment observed the physiological effects of the sustained release of rhTGF-β_3_ from the *ex vivo* experiments using human osteochondral fragments. In the future, the rhTGF-β_3_-loaded EMLDS will be tested in real cartilage environments in order to assess its effect in cartilage repair while considering the presence of synovial fluids and vessels.

Regarding the results obtained in this study, even though rhTGF-β_3_ was found to be released from the ECM multilayer surface, not its side, structures such as pores from which the protein might be released (based on diffusion through the ECM multilayer surface) have yet to be identified. Accordingly, further research on defining the ECM membrane structure and the optimal release conditions through variations in the multilayer thickness should be considered. These results could potentially be extended into various fields, such as skin and ophthalmologic diseases.

## Conclusion

We developed a novel DDS that uses EMLDS that was fabricated through cultivating porcine chondrocytes. The ECM multilayer presented here was demonstrated to be useful for cartilage repair in response to the released rhTGF-β_3_. The results demonstrated that the sustained release of rhTGF-β_3_ was observed over a prolonged period of time *in vitro* and that the released rhTGF-β_3_ maintained its structural stability as well as its biological activity. Successful cartilage repair was also seen when using rabbit MSCs in an *in vivo* model and rabbit chondrocytes and MSCs in *ex vivo* models were responsive to rhTGF-β_3_ released from the EMLDS. Therefore, we conclude that the EMLDS could be a useful drug delivery system for cartilage repair.
